# Supplementary material for: Contact network analysis of Covid-19 in tourist areas——Based on 333 confirmed cases in China
Source: PLoS One. 2021 Dec 14;16(12):e0261335. doi: 10.1371/journal.pone.0261335 (PMC8670701; doi:10.1371/journal.pone.0261335)
Supplement: Attachment — Submitted filename: 20210729-Response Letter.docx [file pone.0261335.s001.docx]

**Authors’ Response**

Dear Editor and Review,

Thank you very much for the helpful comments that you and the reviewer provided on our manuscript. We are very pleased with the detailed modification suggestions and grateful to have the opportunity to refine our paper on these valuable suggestions. As you will see, we have addressed all the issues raised, which are highlighted in yellow in the manuscript and detailed below point by point. We look forward to your rechecking the paper and making other valuable suggestions for us.

Thank you once more for your constructive feedback and tireless patience. We hope that the refined manuscript meets your expectations.

Yours sincerely,

Authors of PONE-D-21-13537

**Main comments:** Please ensure that you refer to Figure 9 in your text as, if accepted, production will need this reference to link the reader to the figure.

**Our response:** Thank you very much for your attentiveness. We have checked the text and corrected this typo to Figure 8.

**Main comments:** As stated in the short summary of my review, the authors have repeatedly made statements on policy which are not justified by the scientific content of the paper. The authors have studied the epidemiological characteristics of a relatively small number of cases and analysed the underlying contact network, and have not carried out any mechanistic modelling of control measures or interventions. I am not suggesting that the authors should be carrying out mechanistic modelling, but they need to remove, or at least qualify, some of their statements on policy.

**Our response:** Thanks for your suggestions. We have removed all statements on policy because we can’t do any intervention.

**Main comments:** In their conclusion section on page 21, the authors say “It shows that prevention and control measure such as quarantine are effective”. First of all, it is not entirely clear what the “It” here is – I assume they are referring to their complete analysis, but it would be good to make this clear. More importantly, the only detailed reference to quarantine measures comes in their analysis of the Yunan transmission network on page 16, where they say “these clusters did not form larger clusters in the late stage, indicating that the infected people were taken medical treatment or quarantined in time”. There appears to be some circular reasoning at work here: the authors infer that small cluster size has been caused by quarantining without explaining why they have made this inference, and then later argue that their analysis supports the use of quarantine. However, based on the evidence provided one could (very facetiously) propose any mechanism one wanted to for the small cluster size, and then later argue that the analysis supports policies involving this mechanism.

**Our response:** According to your suggestions, we removed related discussion on policy measures. We note that our findings can be used as a reference in the policy process, but it is clear that specific policies require a combination of multiple data sources.

**Main comments:** Overall I recommend that the authors completely rewrite all of the sections of their paper which talk about policy interventions. While I do not necessarily disagree with any of these statements, I do not believe that the analysis conducted in the paper is at all sufficient to support the very confident recommendations which the authors make. A justified policy recommendation would need to state the likely impact of the policy, and the work presented here is not capable of predicting such impacts. A more tentative discussion of policy, outlining the ways the authors’ analysis could support a policy response, would be very welcome, but the sweeping recommendations made in the current manuscript would require either a mechanistic modelling study or a direct comparison of data from region simplementing different policies to be justified.

**Our response:** We have completely rewritten the section on policy. We did not revisit the effectiveness of the policy because this was not possible in the absence of interventions and regional comparisons. We mainly discussed the fact that epidemic prevention departments can quickly analyze case texts, integrate case data, map networks, and make them available to the public after anonymization in the case of similar disease outbreaks. This can give the public a clear picture of development of the outbreak.

**Main comments:** In their introduction, the authors state “This virus spreads via respiratory droplets and close proximity interactions (CPIs), with an incubation period of 1-14 days.“ While there has been some controversy on this point I believe it is now well-established that COVID-19 can be spread through airborne transmission (I have provided a reference for this at the end of this review, but I recommend the authors seek out some more references to get a wider view of the evidence on this topic). This transmission pathway is obviously less determined by human social networks than the CPI and droplet components. While a network analysis is still extremely worthwhile, it would be good to point out the limitations implied by airborne transmission; for instance, infection can potentially spread across large rooms or between neighbouring rooms connected by a ventilation system, meaning the index and secondary case may not necessarily have directly interacted and so will not necessarily belong to as hared social contact network.

**Our response:** In addition to the literature given by the reviewer, we also refer to some other literature related to airborne transmission. In the introduction section, we added airborne as a main transmission pathway. In the conclusion section, we added a discussion about the mode of transmission. Airborne transmission makes network analysis cannot depict the full transmission as you pointed out since virus can transmit between two strangers in different space, and the virus transmission should be discussed in the context of geographical location and space. We also put it into the limitation of this study.

**Main comments:** On page 6, the authors introduce some network statistics, stating “These are the three specific network indicators that we used to depict the postion of patients in the network”. I am not sure that “depict” is the right word here. These statistics really act to quantify the patients’ positions, and I would like to see this sentence adjusted accordingly. As an aside, I note here that “position” is misspelt.

**Our response:** Thank you very much for your suggestions for corrections. We have adjusted “depict” to “measure” and modified the misspelt of “position”. New sentence is as: “These are the three specific network indicators that we use to measure the position of patients in the network.”

**Main comments:** The definition of betweenness centrality on page 6 is incomplete. In their verbal explanation the authors say “The betweenness centrality of the patient i is the ratio of the geodesic passing through patient i and connecting patients j and k to the total number of geodesic between patients j and k.” This clearly isn’t acorrect description; based on the description given on Wikipedia, I believe this should be the sum of all such ratios over all distinct pairs of j and k. In their algebraic formula they do not define the term b_jk(i). If this term is supposed to be the ratio, the authors should state this. If it is just meant to be the number of geodesics passing through I, then they also need to add an appropriate denominator to their formula.

**Our response:** Thank you very much for your suggestions. We have revised the calculation formula of the betweenness centrality by adding an appropriate ratio to our formula to make it clearer and more explicit.

**Main comments:** Similarly, the authors do not provide any indication of how to calculate Pagerank index. The authors need to either state how it can be calculated, or provide an appropriate reference. The Brin and Page 1998 paper does not actually include an explicit formula for Pagerank and so is not sufficient here. The authors also mention “patient j’s ego network size” in this section, but do not explain what an ego network is. For publication, a definition of the ego network needs to be provided.

**Our response:** Thank you very much for your corrections. We have stated how Pagerank index can be calculated and provide an appropriate reference. And we also have provided a definition of the ego network. In our study, the patient j’s ego network size means the number of patients connecting with patient j.

**Main comments:** The description of the network component on page 7 is a bit confusing: “Network component means that within it, each patient has a network path reach with others, it may be a direct tie or an indirect tie”. I think what the authors mean is that all members of a network component can be connected by a well-defined path. It would be good to reword this sentence to make its meaning clearer.

**Our response:** Thank you very much for your suggestions. We have corrected the description of the network component on page 7 to make its meaning clearer. Network component means that within it, each patient has a network path reach with others, it may be a direct tie or an indirect tie, all members of a component can be connected by a well-defined path.

**Main comments:** In Figure 1 on page, no indication is given of what the solid line corresponds to. I assume this is a distribution which has been fitted to the data. The authors do not actually appear to use this fitted distribution anywhere in the paper, and no a priori reasoning is given for why the unimodal distribution they have attempted to fit should give a good approximation of the age distribution of cases. In practice the age distribution of cases will depend on a range of factors including the age distribution of the underlying population, the contact patterns within that population, and age-stratified heterogeneities in behaviour and physiology. This complex combination of factors means that a simple parametric distribution is unlikely to give a good explanation of the data, and so I do not think there is anything to be gained from fitting to such a distribution. I therefore recommend that the authors either remove this line from the plot, or provide a description of their fit and a compelling explanation as to why they have carried it out. The choice of bins and axis ticks in Figure 1 also needs to be changed. In the body text they refer to five-year intervals, their bars appear to be defined according to four-year intervals, and the axis ticks are at five year intervals starting at 3. The figure needs to be redrawn with five-year bins starting at zero, and matching axis ticks. In the body text describing Figure 1 the authors say “The frequency distribution of patients’ age is the left-skewed distribution”. I believe they mean that the frequency distribution is left-skewed, since “the left-skewed distribution” is not any specific probability distribution.

**Our response:** Thank you very much for your correction. We have redrawn Figure 1 by removing the solid line and changing axis ticks into five-year intervals starting at zero. We also revised the “left-skewed distribution” sentence: “The frequency distribution of patients’ age is left-skewed (see Figure 1).”

**Typos and minor comments:** Abstract - “The spread of virus is highly related to the stucture of human network.” should be “The spread of virus is highly related to the structure of human networks.”

**Our response:** Thank you very much for your corrections. We have changed this sentence in the abstract as: “The spread of virus is highly related to the structure of human networks”.

**Typos and minor comments:** “There are more male patients than the females in these two districts, most are imported cases, with an average age of 45 years” should be “There are more male patients than the females in these two districts and most are imported cases, with an average age of 45 years”.

**Our response:** Thank you very much for your corrections. We have modified the sentence into “There are more male patients than the females in these two districts and most are imported cases, with an average age of 45 years”.

**Typos and minor comments:** “There is no super spreader in the network” – since outbreaks can have more than one super spreader, this should really say “There are no super spreaders in the network”.

**Our response:** Thank you very much for your corrections. We have revised the sentence into “There are no super spreaders in the network”.

**Typos and minor comments:** Page 2: “Coronavirus Disease 2019 (Covid-19) has outbroken in many countries around the world and drew widespread global attention” should be “Coronavirus Disease 2019 (Covid-19) has broken out in many countries around the world and drawn widespread global attention”

**Our response:** Thank you very much for your corrections. We have amended the sentence into “Coronavirus Disease 2019 (Covid-19) has broken out in many countries around the world and drawn widespread global attention”.

**Typos and minor comments:** Page 3: “Population is generally susceptible.” This statement is a bit vague. Something like “There is little or no pre-existing immunity in most populations”, with an appropriate citation, would be more specific.

**Our response:** Thank you very much for your corrections. We have revised the sentence into “According to the joint investigation report of COVID-19 released by China and World Health Organization in February 2020, there is little or no pre-existing immunity in most populations”.

**Typos and minor comments:** Page 5: “Pior research showed that the immunization strategy based on the actual contact network is better than the random immunization strategy ( Eubank et al., 2004).” While I am not familiar with the Eubank et al. paper, a network-based immunisation strategy can still be random in the sense that each individual has a certain probability of being vaccinated, and I expect the authors mean uniform random immunisation (i.e. each individual has the same probability of being immunised) rather than just random immunisation. The authors have also misspelt “prior” at the start of this sentence.

**Our response:** Thank you very much for your corrections. We do focus on uniform random immunization rather than just random immunization, so we clarified the expression and modified misspelt word as following: “Prior research showed that the immunization strategy based on the actual contact network is better than the uniform random immunization strategy.”

**Typos and minor comments:** Pages 6-7: The list of network statistics (degree centrality, betweenness centrality, Pagerank index) is currently presented as three paragraphs, with the first sentence of each being the name of the statistic. Depending on what the journal’s formatting requirements allow, I would rather see this presented in either a more explicit list form (i.e. with bullet points or at least bold item headings) or as “normal” text (i.e. “The first statistic we consider is…. Our next statistic is…. Finally, we consider….”).

**Our response:** Thank you very much for your corrections. We have revised the first sentence at the beginning of the paragraph on Pages 6-7 into unified and standardized format: “The first statistic we consider is degree centrality.” “Our next statistic is betweenness centrality.” “Then we consider Pagerank index.”

**Typos and minor comments:** Page 7: “Over 80% of patients were infected out of the province, which means there are more imported cases.” While the meaning here is clear, this is slightly poor wording, and should be amended to something like “Over 80% of patients were infected out of the province, which means that most cases are imported.”

Table starting on page 7 spills over to the next page and should be reformatted.

**Our response:** Thank you very much for your corrections. We have amended the sentence on Page 7 into “Over 80% of patients were infected out of the province, which means that most cases are imported”. We also reformatted Table 1 for the spilling over problem.

**Typos and minor comments:** Page 9: “Hainan Province, where is a major pure tourism area in China” should be “Hainan Province, which is a major pure tourism area in China”

Table 2 on page 11: the column labelled “Case number” should be labelled “Number of cases”.

**Our response:** Thank you very much for your corrections. We have revised the sentence into “Hainan Province, which is a major pure tourism area in China” on Page 9. And we also have changed the column of Table 2 on page 11 into “Number of cases”.

**Typos and minor comments:** Tables 3 and 4 have final column labelled “Maximus value”, which should be “Maximum value”.

**Our response:** Thank you very much for your corrections. We have modified the word “Maximus” into “Maximum” in Tables 3 and 4.

**Typos and minor comments:** Page 22: “In general, most of the researches on the COVID-19 epidemic…” should be “In general, most of the research on the COVID-19 epidemic…”.

**Our response:** Thank you very much for your corrections. We have modified the sentence into “In general, most of the research on the COVID-19 epidemic…”.

**Data availability** – while the authors have provided a link to a repository containing their data and code, I was unable to access it. Under the “Files” tab on the repo website I got a message saying “Loading files” which did not resolve itself after >10 minutes of waiting. Because I can not see the code and the authors have also not provided a complete methodology for their calculation of betweenness centrality and Pagerank index, I can not confirm that their analysis has been carried out correctly.

**Our response:** We are sorry about the improper link. We re-uploaded our research data on a new data repository ICPSR. Please access the deposit workspace at: https://www.openicpsr.org/openicpsr/project/145901/version/V2/view. The data and code are in a Stata format and the network file is in a Gephi file, you may need relevant software to open them. Apologize again for the inconvenience we have brought to your work.

**Our response:** Moreover, we asked a copy editor to help improve our language. Thank you again for your very detailed and extremely constructive comments, they help improve this paper a lot. We would not have got a high-quality paper without these sincere suggestions. We look forward to hear more about your feedback.
