# Supplementary material for: Contact network analysis of Covid-19 in tourist areas——Based on 333 confirmed cases in China
Source: PLoS One. 2021 Dec 14;16(12):e0261335. doi: 10.1371/journal.pone.0261335 (PMC8670701; doi:10.1371/journal.pone.0261335)
Supplement: Attachment — Submitted filename: 20210924-Response Letter.docx [file pone.0261335.s002.docx]

**Authors’ Response**

Dear Editor and Reviewer,

Thank you very much for the targeted and constructive comments that you and the reviewer provided on our manuscript. We are very pleased with the detailed modification suggestions and grateful to have another opportunity to refine our paper. As you will see, we have addressed all the issues raised again, which are highlighted in yellow in the manuscript and detailed below point by point. We look forward to your rechecking the paper and making other valuable suggestions for us.

Thank you once more for your constructive feedback and tireless patience. We hope that the refined manuscript meets your expectations.

Yours sincerely,

Authors of PONE-D-21-13537

**Main comments:** The only aspect of the paper which I feel is still insufficient Is the section on Pagerank index on pages 6 and 7. While this section is more detailed than it was previously, it still isn’t totally clear how Pagerank is calculated based on the details the authors provide. In particular, the authors do not define B_i, the set which they sum over in the formula at the top of page 7. However, given the authors have now provided a reference (Easley and Kleinberg 2010) which includes a detailed definition of Pagerank, I think it will be sufficient to give a qualitative description and point the reader to the reference for full detail. This description just needs to cover the basic idea that Pagerank measures how likely one is to arrive at a given node by moving randomly around a network, and that it is calculated iteratively.

**Our response:** Thank you very much for your attentiveness. We gave the define B_i in this section, where B_i is the set containing all nodes linking to the focal node j. We also have added a qualitative explanation of Pagerank that it measures how likely one is to arrive at a given node by moving randomly around a network, and it is calculated iteratively. We remind our readers that they can find the detailed calculation procedure in the book by Easley and Kleinberg.

**Main comments:** While Figures 6 and 7 are useful for visualising the epidemic network, they are currently a bit small. Depending on the journal’s formatting requirements, it would be good to make these figures larger, possibly by rearranging each one into a 3x1 column of plots rather than the current 1x3 rows.

**Our response:** Thanks for your suggestions. We have rearranged the plot of the pictures again to show more clear details of the contact network. Please see Figures 6 and 7.

**Minor grammar/typo notes**

**Minor comments:** Abstract: “The spread of virus is highly related to the structure of human networks” should be “The spread of viruses is highly related to the structure of human networks”, or, since this holds for other types of pathogen, “The spread of infectious diseases is highly related to the structure of human networks”.

**Our response:** Thank you very much for your suggestions for corrections. We have revised the sentence as “The spread of infectious diseases is highly related to the structure of human networks”.

**Minor comments:** Abstract: “There are more male patients than the females in these two districts…” should be “There are more male patients than females in these two districts…”.

**Our response:** Thank you very much for your suggestions for corrections. We have deleted the word “the” and revised the sentence as “There are more male patients than females in these two districts…”.

**Minor comments:** Top of page 5: “This study collects detailed information of patients diagnosed with COVID-19…” should be “This study collects detailed information about patients diagnosed with COVID-19…”.

**Our response:** Thank you very much for your corrections. We have adjusted the sentence as “This study collects detailed information about patients diagnosed with COVID-19…”.

**Minor comments:** Bottom of page 6: “By many times of iterations, the Pagerank value of each node in the network converge to a limiting value”.

**Our response:** Thank you. We have corrected the sentence as “The Pagerank value of all nodes in the network converge to limiting values as the number of iterations goes to infinity”.

**Minor comments:** Top of page 10: “We also summarized the number of days that symptom had emerged”. I think the authors here mean “We also summarized the number of days between arrival in Hainan or Yunan and emergence of symptoms”, although the authors should confirm that this is indeed the intended meaning.

**Our response:** Thank you very much for your correction. We have clarified the sentence as “We also summarized the number of days between arrival in Hainan or Yunan and emergence of symptoms”.

**Minor comments:** Page 15: “However, these clusters did not form larger clusters in the late stage, indicating that most infected people were taken medical treatment or quarantined in time” should be “However, these clusters did not form larger clusters in the late stage, indicating that most infected people were given medical treatment or quarantined in time”

**Our response:** Thank you very much. We have changed this sentence as: “However, these clusters did not form larger clusters in the late stage, indicating that most infected people were given medical treatment or quarantined in time”.

**Minor comments:** Page 20: “When these information be quickly collected…” should be “When these information are quickly collected…”

**Our response:** Thank you very much for your corrections. We have modified the sentence into “When these information are quickly collected…”.

**Minor comments:** Page 20: “In general, most of the research on the COVID-19 epidemic focus on the epidemiological characteristics of confirmed patients” should be “In general, most of the research on the COVID-19 epidemic has focused on the epidemiological characteristics of confirmed patients”

**Our response**: Thank you. We have revised the sentence into “In general, most of the research on the COVID-19 epidemic has focused on the epidemiological characteristics of confirmed patients”.

**Minor comments:** Page 21: “Because of limited date” should be “Because of limited data”.

**Our response:** Thank you. We corrected the mistakes in the sentence.

**Our response:** Moreover, we checked the full manuscript again and corrected some other grammatical errors. Thank you again for your very detailed and extremely constructive comments, they help improve this paper a lot. We would not have got a high-quality paper without these sincere suggestions. We look forward to hear more about your feedback.
