# Supplementary material for: Contact network analysis of Covid-19 in tourist areas——Based on 333 confirmed cases in China
Source: PLoS One. 2021 Dec 14;16(12):e0261335. doi: 10.1371/journal.pone.0261335 (PMC8670701; doi:10.1371/journal.pone.0261335)
Supplement: Attachment — Submitted filename: 20211029-Response Letter-R3.docx [file pone.0261335.s003.docx]

**Authors’ Response**

Dear Editor and Reviewer,

Thank you very much for the targeted and constructive comments that you and the reviewer provided on our manuscript. We are very pleased with the detailed modification suggestions and grateful to have another opportunity to correct errors and refine our work. As you will see, we have addressed all the issues raised again, which are highlighted in yellow in the manuscript and detailed below point by point. We look forward to your rechecking the paper and making other valuable suggestions for us.

Thank you once more for your constructive feedback and tireless patience. We hope that the refined manuscript meets your expectations.

Yours sincerely,

Authors of PONE-D-21-13537

**Main comments:** In the abstract the authors state that there are more male than female patients, whereas Table 1 (pages 7 to 8) and the accompanying text on page 7 show that there are 142 female patients to 140 male patients. The abstract text should be corrected accordingly.

**Our response:** Thank you very much for your attentiveness. We have compared the data in Table 1, that is, the number of female patients is more than that of male patients, and revised the expression in the abstract.

**Main comments:** Further, on page 9 the authors say “In the data of Hainan Province, except for January 27, January 28, and February 9, the average age is stable at 40-60 years old, that is, these cases are older people with a weakened immune system”. This is a fairly sweeping statement. First of all, the authors do not indicate what the mean age of the underlying population is – if the mean population age is in the 40-60 years old range, then this average case age is what one would expect with no underlying age structure to transmission or symptom intensity. If 40-60 is actually older than the baseline population average, then there is still no direct evidence that the high number of cases in older individuals is caused by older individuals having weakened immune systems. This is simply one hypothetical explanation, and the authors should present it as a possibility rather than a certainty.

**Our response:** Thanks for your suggestions. In this section, we increased the proportions of people aged 60 and above among the cases in Yunnan and Hainan Province, and compared them with the proportions of people of this age in these two provinces shown in the seventh National Census of China. By comparing the difference of the proportion of the elderly in the patients and the census data of the whole province, we find that the proportion of the elderly in the patients is higher, which means that the elderly may have a larger proportion in the confirmed patients due to weak immunity and other reasons. Therefore, we revised the statement in the paper to “Tourism in Yunnan Province is typically less concentrated towards older age groups. In Yunnan Province, 21.01% of the cases are aged 60 years and above, which exceeds the proportion of the local population aged 60 years and above in the total population (14.91%, China the seventh National Census). In Hainan Province, 32.72% of the cases are aged 60 years and above, which is greater than the proportion of this age group in the total population (14.65%, China the seventh National Census).”.

**Main comments:** On page 22 the authors then say “At the late stage, cases

are mostly elderly people who have a weak immune system, poor mobility ability, and weak transmission ability”. This should be modified to clarify that these are all risk factors associated with old age, but that not every case will exhibit all of these risk factors (and some may exhibit none of them).

**Our response:** Thanks for your suggestions. We have modified to clarify that higher age is likely to cause patients to who have a weak immune system, poor mobility ability, and weak transmission ability.

**Minor grammar/typo notes**

**Minor comments:** Page 1: “On January 30, 2020, The World Health Organization declared the epidemic as a Public Health Emergency of International Concern.” – the (newly added) word “as” is not necessary here.

**Our response:** Thank you very much for your suggestions for corrections. We have deleted the word “as” in the sentence and revised the sentence as “On January 30, 2020, The World Health Organization declared the epidemic a Public Health Emergency of International Concern”.

**Minor comments:** Page 4: “Human social network is heterogeneous” should be “Human social networks are heterogeneous”.

**Our response:** Thank you very much for your suggestions for corrections. We have revised the sentence as “Human social networks are heterogeneous”.

**Minor comments:** Page 5: “The specific steps of text mining and coding are as following:” should be “The specific steps of text mining and coding are as follows.” Note that since that the sentence is followed by a paragraph break rather than a bulleted list, the authors should replace the semi colon with a full stop.

**Our response:** Thank you very much for your corrections. We have adjusted the sentence as “The specific steps of text mining and coding are as follows”.

**Minor comments:** Page 6: “PageRank measures how likely one is to arrive at a given node by moving randomly around a network, and that it is calculated iteratively” should be “PageRank measures how likely one is to arrive at a given node by moving randomly around a network, and is calculated iteratively”.

**Our response:** Thank you. We have corrected the sentence as “PageRank measures how likely one is to arrive at a given node by moving randomly around a network, and is calculated iteratively”.

**Minor comments:** Page 7: “The specific formula is as following” should be “The specific formula is as follows”.

**Our response:** Thank you very much for your correction. We have clarified the sentence as “The specific formula is as follows”.

**Minor comments:** Page 7: “A component means that, within in it, all members can be conneted by a well-defined path.” – “connected” is misspelt.

**Our response:** Thank you very much. We corrected the misspelt word in the sentence.

**Minor comments:** Page 9: “One possible reason for the age gap might be that tourist industry is the pillar industry in Hainan Province” should be “One possible reason for the age gap might be that tourism is the pillar industry in Hainan Province”

**Our response:** Thank you very much for your corrections. We have modified the sentence into “One possible reason for the age gap might be that tourism is the pillar industry in Hainan Province”.

**Minor comments:** Page 9: “As for Yunnan Province, though it is also a tourist attraction, it attracts people at all ages. Besides, Yunnan is one of the biggest labor exporters in China, these returning migrant workers, who are mostly are young, also contributes to the lower the average age of Yunnan.” The phrasing of the first sentence feels slightly informal for a scientific paper. Something like “Tourism in Yunan Province is typically less concentrated towards older age groups” would be a bit more fitting. I do not find the second sentence at all convincing – being a net exporter of migrant labour will make the average age higher, not lower. Returning workers will bring the age profile back up to the pre-migration average but will not increase it past that level. Unless I have seriously misunderstood the reasoning here, this statement should be removed.

**Our response**: Thank you. We have revised the sentence into “Tourism in Yunan Province is typically less concentrated towards older age groups”. We also removed the impertinent statement in the paragraph

**Minor comments:** Pages 11-12: “For example, cases No. 73 and No. 87 in Hainan Province crossed districts and counties for about 8 times” should just be “For example, cases No. 73 and No. 87 in Hainan Province crossed districts and counties about 8 times”.

**Our response:** Thank you. We corrected the mistakes and revised the sentence into “For example, cases No. 73 and No. 87 in Hainan Province crossed districts and counties about 8 times”.

**Minor comments:** Page 19: “maximum” is misspelt as “maximun”.

**Our response:** Thank you. We corrected the mistake in the sentence and spelled the word “maximun” again.

**Minor comments:** Page 24: “In such a condition” should be “Under such conditions”.

**Our response:** Thank you. We corrected the mistake and revised the sentence into “Under such conditions”.

**Our response:** Moreover, we checked the full manuscript again and corrected some other grammatical errors. Thank you again for your very detailed and extremely constructive comments. They help us to improve this paper a lot. We would not have got a high-quality paper without these sincere suggestions. We look forward to your feedback.
